# System resilience distribution identification and analysis based on performance processes after disruptions

**DOI:** 10.1371/journal.pone.0276908

**Published:** 2022-11-03

**Authors:** Yeqing Song, Ruiying Li

**Affiliations:** 1 School of Reliability and Systems Engineering, Beihang University, Beijing, China; 2 Science and Technology on Reliability and Environmental Engineering Laboratory, Beijing, China; Universidad Rey Juan Carlos, SPAIN

## Abstract

Resilience is a system’s ability to withstand a disruption and return to a normal state quickly. It is a random variable due to the randomness of both the disruption and resilience behavior of a system. The distribution characteristics of resilience are the basis for resilience design and analysis, such as test sample size determination and assessment model selection. In this paper, we propose a systematic resilience distribution identification and analysis (RDIA) method based on a system’s performance processes after disruptions. Typical performance degradation/recovery processes have linear, exponential, and trigonometric functions, and they have three key parameters: the maximum performance degradation, the degradation duration, and the recovery duration. Using the Monte Carlo method, these three key parameters are first sampled according to their corresponding probability density functions. Combining the sample results with the given performance function type, the system performance curves after disruptions can be obtained. Then the sample resilience is computed using a deterministic resilience measure and the resilience distribution can be determined through candidate distribution identification, parameter estimation, and a goodness-of-fit test. Finally, we apply our RDIA method to systems with typical performance processes, and both the orthogonal experiment method and the control variable method are used to investigate the resilience distribution laws. The results show that the resilience of these systems follows the Weibull distribution. An end-to-end communication system is also used to explain how to apply this method with simulation or test data in practice.

## Introduction

Ideally, systems are designed with the expectation that they will run smoothly and sustainably. However, a system often faces various disruptions including an external disruption (such as natural disaster and malicious attack) or the internal failure of the system itself. Disruption will reduce the system performance or cause system failure, or even a domino effect. Thus, a system should have the ability to withstand disruption and return to a normal state quickly, i.e., resilience, to prevent and minimize the losses from disruption.

The term “resilience” originates from the Latin word *resiliere*, which means to bounce back [1]. It was first proposed in ecology and was later applied to other fields such as communications systems and power systems. So far, the study of resilience has attracted great attention. Some scholars have concentrated on how to quantify system resilience [2–5], and some have discussed how to maintain and enhance system resilience [6–9]. Researchers also found that resilience was an internal property of a system, and claimed that it should be considered in the early design and development stages of systems [10, 11].

As a random variable due to the randomness of both the disruption and the system’s response to it, resilience distribution characteristics are the basis for system resilience design and analysis. These characteristics are useful in understanding system resilience from a statistical perspective, and they can be further used in resilience index determination, resilience test program design, and resilience assessment model selection. For different distribution types, the corresponding resilience index, test program and assessment model are different. For example, for system resilience with normal distribution and exponential distribution, when engineers determine the resilience index (e.g., the expected resilience), they will consider whether the resilience distribution is symmetric or skewed. In industry, for systems with similar resilience distributions, unified test and assessment specifications can be formulated for the convenience of engineers. As is known, for system characteristics with different types of distributions, the sample sizes and the assessment methods are very different.

However, to the best of our knowledge, few studies have analyzed resilience distribution, and only some research has provided resilience analysis results using distribution forms. For example, Ouyang et al. [12] calculated the resilience distribution of a power transmission grid for different hazard scenarios. Pant et al. [13] obtained the cumulative density function (CDF) of a container terminal’s resilience using a simulation for given recovery orders and recovery probability distributions. Ba-Alawi et al. [14] conducted a resilience assessment of the membrane bioreactor in a wastewater treatment plant based on the performance curves, with the assumption that the distribution of the failure data followed a lognormal distribution. Zinetullina et al. [15] proposed a quantitative resilience assessment for chemical process systems with normally distributed variables such as timing and precision. On the other hand, some researchers analyzed the resilience laws of complex networks such as transportation systems, water supply systems, and electric power systems. For example, Orosz et al. [16] explored the relationship between resilience and the minimal production flow rate of a process network. Mou et al. [17] found that the resilience of a crude oil transportation network decreased at a steady rate during random attacks and decreased sharply during deliberate attacks. They also found that the density and centrality of the network were negatively correlated with resilience, while the connectivity and size of the network were positively correlated with resilience. Table 1 summarized these related works, and one can find that how to analyze the system resilience distribution is still a problem.

**Table 1:** Comparison of related works.

Source	Disruption type	Application area	Comparisons
Ouyang et al. [12]	Hurricane and random hazards	Power and other infrastructure systems	The distributions of hazard occurrence rate and intensity are modeled with historical data, and the system’s resilience distributions was obtained by simulation. The resilience distribution type was not further analyzed.
Pant et al. [13]	Terminal closure and crane outage	Container terminals	The randomness of repair arrival rate is considered, and the system resilience distribution is analyzed. Also, the resilience distribution type was not further analyzed.
Ba-Alawi et al. [14]	Basic events that may cause system failure	Wastewater treatment plant	The failure time distribution is considered, and the system resilience is computed for four different seasons. The resilience distribution is not analyzed.
Zinetullina et al. [15]	Unprecedented disruption	Chemical process systems	The randomness of the failures is considered, and the system’s resilience profile can be obtained as a probabilistic and time-dependent evolution of a system’s functionality state. The resilience distribution is not analyzed.
Orosz et al. [16]	Expected or unexpected failures	Processing systems	The system resilience is analyzed with different redundancy configurations, and how redundancy effects on system resilience is studied.
Mou et al. [17]	Random and intentional attacks	Crude oil transportation network	How attack type and network topology characteristics effect on network resilience is studied.

In this study, we first summarize the system’s possible behavior after a disruption and propose our resilience distribution identification and analysis (RDIA) method. Then, using this method, the resilience distribution for some typical performance processes is analyzed. The results show that the resilience of these systems follows the Weibull distribution.

## Problem description

A system’s resilience is determined by its possible response after a disruption. To find the resilience distribution, we analyze the system’s response first.

**Fig 1:**
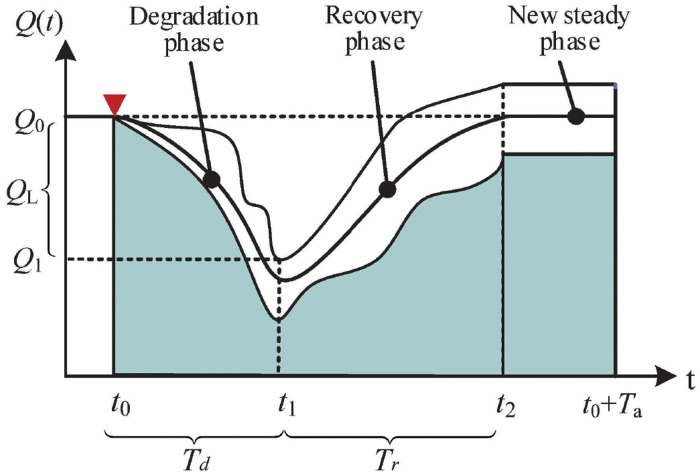
A typical performance process after a disruption.

After a disruption, the system may experience three main phases: i) degradation phase, ii) recovery phase, and iii) new steady phase, as shown in Fig 1. In the beginning, the system runs normally. Then, a disruption occurs at time *t*_0_, and the system performance begins to degrade and enters the degradation phase. Then the recovery actions are taken to cope with the disruption and the system begins to recover and enters the recovery stage. The performance degradation time and the recovery time are denoted as *T*_*d*_ and *T*_*r*_, respectively, and the maximum performance degradation is denoted as *Q*_*L*_.

**Fig 2:**
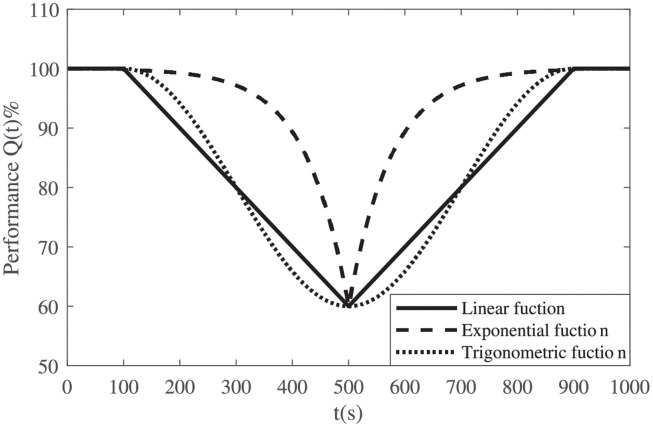
Typical system performance process functions.

The system’s ability to absorb, adapt, and recover from the disruption determines its performance degradation and recovery process. Cimellaro et al. [18] proposed three types of performance recovery functions, i.e., linear, exponential, and trigonometric functions. Similarly, these functions were also used to describe the performance degradation processes (see [19–21]). For those systems that can be fully recovered, these functions are as follows:

1. Degradation function (from time *t*_0_ to *t*_1_)
  $$\begin{array}{c} Q_{d}\left(t\right)=\{\begin{array}{c} 1-Q_{L}(\frac{t-t_{0}}{T_{d}}),\;\left(\text{linear}\right)\\ 1-Q_{L}\frac{\text{exp}(-b\frac{T_{d}-t+t_{0}}{T_{d}})-\text{exp}\left(-b\right)}{1-\text{exp}(-b)},\;\left(\text{exponential}\right)\\ 1-0.5Q_{L}[1-\text{cos}(\pi \frac{t-t_{0}}{T_{d}})],\;\left(\text{trigonometric}\right) \end{array} \end{array}$$ (1)
2. Recovery function (from time *t*_1_ to *t*_2_)
  $$\begin{array}{c} Q_{r}\left(t\right)=\{\begin{array}{c} 1-Q_{L}(1-\frac{t-t_{1}}{T_{r}}),\;\left(\text{linear}\right)\\ 1-Q_{L}\frac{\text{exp}(-b\frac{t-t_{1}}{T_{r}})-\text{exp}\left(-b\right)}{1-\text{exp}(-b)},\;\left(\text{exponential}\right)\\ 1-0.5Q_{L}[1+\text{cos}(\pi \frac{t-t_{1}}{T_{r}})],\;\left(\text{trigonometric}\right) \end{array} \end{array}$$ (2)

where *b* represents the scale parameters in the exponential function and determines the extent to which the exponential function deviates from linearity (*b* > 0). The three types of performance process functions are shown in Fig 2, and they can be used in the following situations:

1. The linear function is suitable for systems with a constant performance change rate. It is generally used when there is limited information regarding emergency preparation, available resources, and the system’s response to the disruptions.
2. The exponential function can be applied for systems with a ‘first slow then fast’ performance degradation process or with a ‘first fast then slow’ recovery process. A larger *b* indicates a more significant deviation from the linear function and a more obvious change rate.
  - An exponential degradation process indicates that the system can resist the disruption at the beginning, and the degradation increases over time because all possible resistance strategies have been used.
  - An exponential recovery process implies that the system’s performance is restored quickly after the recovery action starts, and the speed slows down later. This phenomenon may be caused by a repair sequence in which those actions with a large recovery effect are taken first.
3. The trigonometric function is suitable for systems with slower performance degradation/recovery at both the beginning and the end of the process, but with a faster performance change rate in the middle.
  - A trigonometric degradation process means that the disruption has a slight impact on the system in the initial stage, but its impact increases along with the disruption intensity. At the end of the process, the performance is not easily affected, and the degradation speed slows down.
  - A trigonometric recovery process represents the fact that the system lacks or has limited resources at the beginning. Once the resource is obtained, the recovery speed increases quickly, as those actions that have a large recovery effect on the system are taken first. Therefore, the recovery speed increases at the beginning and then slows down slightly at the end.

There are three key parameters in the performance degradation and recovery process after a disruption, including the maximum performance degradation *Q*_*L*_, the degradation duration *T*_*d*_, and the recovery duration *T*_*r*_. Usually, *Q*_*L*_ is determined by the system redundancy and the disruption severity, and *T*_*d*_ and *T*_*r*_ are affected by the recovery strategies such as resource allocation and repair sequences. Researchers have also analyzed these parameter characteristics. For example, Wang et al. [22] stated that both the instant residual availability and the recovery time of a link in a road network had a confirmed half-normal distribution when a link was randomly disrupted. Ouyang et al. [12] used a power law distribution to model the hazard intensity of a power transmission system in Harris County and applied the normal distribution to model the repair time of failed substations. An investigation made by Carreras et al. [23] showed that the blackout time intervals for power systems approximately satisfied an exponential distribution. Weiss and Rosenthal [24] compared the durations of the supply disruption of an economic order quantity inventory system with a normal distribution and an exponential distribution. Upadhya and Srinivasan [25] applied an exponential distribution for a fighter aircraft’s repair time and log-normal distribution for the logistic delays. Myrefelt [26] found that log-normal distributions best fit the mean time to repair (MTTR) and mean time between failures (MTBF) data for a heating, ventilation, and air conditioning system.

## Method

For the system with the given system response characteristics, we propose a Resilience Distribution Identification and Analysis (RDIA) method, and analyze the system resilience distributions. Our RDIA method makes the following assumptions:

1. The system can finally return to the initial performance after recovery;
2. Both the system performance degradation and the recovery processes are monotonic.

**Fig 3:**
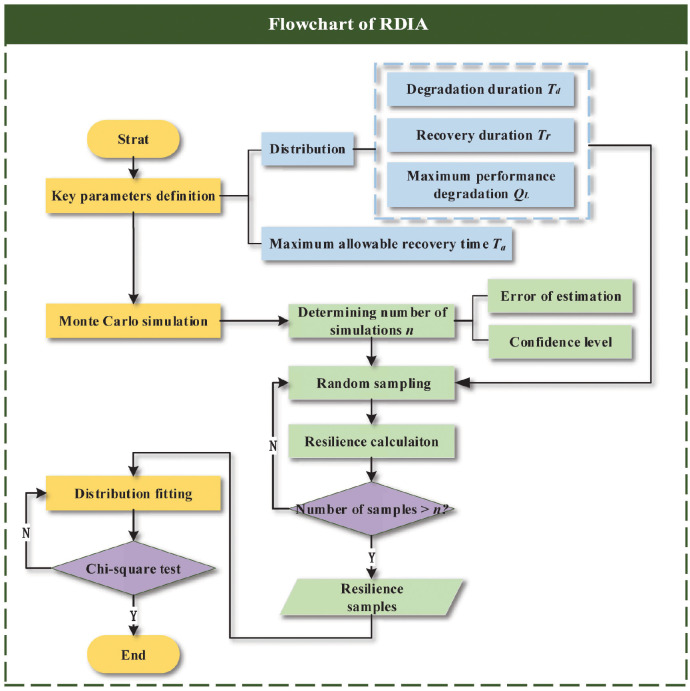
Flowchart of the RDIA method.

Fig 3 expresses the steps of the RDIA method. The specific steps are as follows:

### Resilience samples obtaining

To accurately identify the resilience distribution, the resilience data should be sufficient. In the Monte Carlo simulation, it is assumed that the simulation error is required to be no more than ±*ε* at a confidence level of 1 − *α*. According to the central limit theorem, the number of simulations *n* should satisfy the following equation (see [25, 27]):

$$\begin{array}{c} n\geq {\left(\frac{z_{1-\frac{\alpha }{2}}\sigma }{\varepsilon }\right)}^{2}, \end{array}$$ (3)

If the variance *σ*^2^ is unknown, the sample variance *S* can be taken as an unbiased estimate of the variance. In general, it is recommended that *n* ≥ 1000.

The Monte Carlo method, which uses “frequency” to approximate “probability” with enough experimental data, is applied to obtain the performance behavior samples after the disruption. The steps are as follows:

1. Sampling the key parameters of the performance process after disruptions: n sets of the three key parameters of the system (i.e., *T*_*d*_, *T*_*r*_, and *Q*_*L*_) are sampled according to their distributions.
2. Determining the resilience curve: The resilience curve is generated using the key parameters sampled above and the performance degradation/recovery functions. Then *n* performance processes after disruptions are determined.
3. Obtaining the resilience samples: *n* resilience values are calculated using the resilience measure in Eq 4 (see [27]), i.e.,
  $$\begin{array}{c} R_{D}=\frac{\int_{t_{0}}^{t_{0}+T_{a}}Q(t)dt}{\int_{t_{0}}^{t_{0}+T_{a}}Q_{0}\left(t\right)dt}, \end{array}$$ (4)
  where *Q*(*t*) and *Q*_0_(*t*) represent the system performance at time *t* with and without disruption, respectively, *t*_0_ is the disruption occurrence time, and *T*_*a*_ is the maximum allowable recovery time. This measurement reflects the system’s average performance after a disruption within a certain time period.

### Probability distribution fitting and testing

After obtaining the resilience samples, the probability distribution fitting and testing have the following three steps [28]:

#### Candidate distribution identification

To construct the resilience sample distribution histogram, we first calculate the number of groups as:

$$\begin{array}{c} k=[1+3.3lgn], \end{array}$$ (5)

where *k* is the number of groups, [⋅] indicates rounding, and *n* is the sample size. Then we combine those groups with a sample size of less than five and construct the resilience sample distribution histogram. If the histogram is symmetrically or approximately symmetrically distributed, the resilience samples may obey a normal distribution or a Weibull distribution with a shape parameter between 3 and 4. If the data are right-skewed, an exponential distribution, log-normal distribution, and Weibull distribution should be considered [29]. After possible theoretical distributions are selected, the probability plot can further be used for verification, and the distribution with the best fitting effect (i.e., the points in the probability plot lie approximately on a line) can be selected.

#### Parameter estimation

The methods of parameter estimation mainly include the moment estimation and the maximum likelihood estimation (MLE). The estimation results obtained with the MLE are more significant if the population distribution is known since the posterior information of the samples can be fully utilized. In this study, the MLE is used for the resilience parameter estimation. Letting the distribution of the resilience population be $f(R_{i},\theta_{1},\theta_{2},...,\theta_{m})$, where *θ*_*i*_ is an unknown parameter, and $\left(R_{1},R_{2},...,R_{n}\right)$ are the observed resilience sample values obtained by the Monte Carlo simulation, then, the maximum likelihood function can be written as $L(\theta_{1},\theta_{2},\ldots ,\theta_{m})=\prod_{i=1}^{n}f(R_{i},\theta_{1},\theta_{2},\ldots ,\theta_{m})$. Taking the logarithm, we have *ln*[*L*(*θ*_1_, *θ*_2_, $\ldots ,\theta_{m})]=\prod_{i=1}^{n}ln[f(R_{i},\theta_{1},\theta_{2},\ldots ,\theta_{m})]$. Since the obtained resilience samples are continuous random variables, the maximum likelihood functions are differentiable. Then the likelihood equations can be established as $\frac{\partial \text{ln}L(\theta_{1},\theta_{2},\ldots ,\theta_{m})}{\partial \theta_{i}}=0,\left(i=1,2\ldots m\right)$. By solving this, we can obtain the estimated parameters (*θ*_1_, *θ*_2_, …, *θ*_*m*_).

#### Goodness-of-fit test

Considering the diversity of the resilience sample distributions and the large sample data obtained with the Monte Carlo method, in this study, the chi-squared test method is applied for the goodness-of-fit test. This method uses chi-squared statistics *χ*^2^ (see Eq 6) to represent the deviations between the observed values and the expected distribution. According to the large number theorem, when the samples obey a certain distribution, the number of samples in each group should be close to the number calculated using the theoretical distribution. Therefore, the smaller the chi-squared statistics is, the smaller the deviation is. The chi-squared statistics can be calculated as follows:

$$\begin{array}{c} \chi^{2}=\sum_{i=1}^{k}\frac{{\left(O_{i}-np_{i}\right)}^{2}}{np_{i}}, \end{array}$$ (6)

where *k* is the number of groups, *O*_*i*_ is the number of samples in group *i*, *n* is the sample size, *p*_*i*_ is the probability that sample *X*_*i*_ belongs to group *i* if the distribution assumption holds, *p*_*i*_ = *F*(*x*_*i*_) − *F*(*x*_*i*−1_), and *F*(*x*) is the CDF.

The maximum likelihood estimation is used to replace the unknown parameters in the resilience distribution. The critical value $\chi_{\alpha }^{2}\left(k-m-1\right)$ follows the chi-squared distribution with the degree of freedom *k* − *m* − 1, where *m* is the number of unknown parameters and *α* is the significance level. According to the given significance level *α* and the corresponding quantile of the chi-squared distribution, we can find the critical value $\chi_{\alpha }^{2}\left(k-m-1\right)$ in the chi-squared table. Then, this value can be compared with the chi-squared statistics calculated using Eq 6. If the statistics are less than this critical value, it is considered that the sample observation values obey the assumed distribution, i.e., the resilience distribution is obtained. Otherwise, new distribution types should be considered based on other candidate distributions.

## Experiments and discussions

Using our RDIA method described above, this section discusses the investigation of the resilience distribution of systems with typical performance processes after disruptions. In this study, it is given that:

1. Both the system performance degradation and the recovery processes follow linear, exponential, or triangular functions as Eqs 1 and 2 state.
2. Both the degradation duration *T*_*d*_ and the recovery duration *T*_*r*_ of the system performance follow negative exponential distributions.
3. The maximum performance degradation *Q*_*L*_ follows a discrete uniform distribution.
4. The maximum allowable recovery time *T*_*a*_ is 1000 s.

### Resilience distribution identification and analysis

#### Experiment design

To investigate the resilience distribution of systems with different performance degradation/recovery process functions and parameters, we design five three-level factors experiments according to the *L*18(3^7^) orthogonal array, as shown in Table 2. The performance degradation duration *T*_*d*_ and recovery duration *T*_*r*_ obey the negative exponential distribution with the mean values of 1/λ = 30, 60, 90 seconds. The maximum performance degradation obeys discrete uniform distributions with $P\left(Q_{L}=0.01i\right)=\frac{1}{\text{max}(Q_{L})}$ for *i* = 1, 2, …, max(*Q*_*L*_) and max(*Q*_*L*_) = 30%, 60%, 90%. To facilitate the research, *b* is assumed to be *ln*(200) in the exponential function.

**Table 2:** Experimental design.

No.	Performance process parameters			Performance process functions	
	*Q*_*L*_	*T*_*d*_	*T*_*r*_	*F*_*d*_	*F*_*r*_
1	*P*(*Q*_*L*_ = 0.01*i*) = 0.033, (*i* = 1, 2, …, 30)	1/λ_*d*_ = 30 s	1/λ_*r*_ = 30 s	linear	linear
2		1/λ_*d*_ = 30 s	1/λ_*r*_ = 60 s	exponential	trigonometric
3		1/λ_*d*_ = 60 s	1/λ_*r*_ = 30 s	trigonometric	trigonometric
4		1/λ_*d*_ = 60 s	1/λ_*r*_ = 90 s	linear	exponential
5		1/λ_*d*_ = 90 s	1/λ_*r*_ = 60 s	trigonometric	exponential
6		1/λ_*d*_ = 90 s	1/λ_*r*_ = 90 s	exponential	linear
7	*P*(*Q*_*L*_ = 0.01*i*) = 0.017, (*i* = 1, 2, …, 60)	1/λ_*d*_ = 30 s	1/λ_*r*_ = 30 s	trigonometric	exponential
8		1/λ_*d*_ = 30 s	1/λ_*r*_ = 90 s	linear	trigonometric
9		1/λ_*d*_ = 60 s	1/λ_*r*_ = 60 s	exponential	exponential
10		1/λ_*d*_ = 60 s	1/λ_*r*_ = 90 s	trigonometric	linear
11		1/λ_*d*_ = 90 s	1/λ_*r*_ = 30 s	exponential	trigonometric
12		1/λ_*d*_ = 90 s	1/λ_*r*_ = 60 s	linear	linear
13	*P*(*Q*_*L*_ = 0.01*i*) = 0.011, (*i* = 1, 2, …, 90)	1/λ_*d*_ = 30 s	1/λ_*r*_ = 60 s	trigonometric	linear
14		1/λ_*d*_ = 30 s	1/λ_*r*_ = 90 s	exponential	exponential
15		1/λ_*d*_ = 60 s	1/λ_*r*_ = 30 s	exponential	linear
16		1/λ_*d*_ = 60 s	1/λ_*r*_ = 60 s	linear	trigonometric
17		1/λ_*d*_ = 90 s	1/λ_*r*_ = 30 s	linear	exponential
18		1/λ_*d*_ = 90 s	1/λ_*r*_ = 90 s	trigonometric	trigonometric

#### Procedure and results

**Fig 4:**
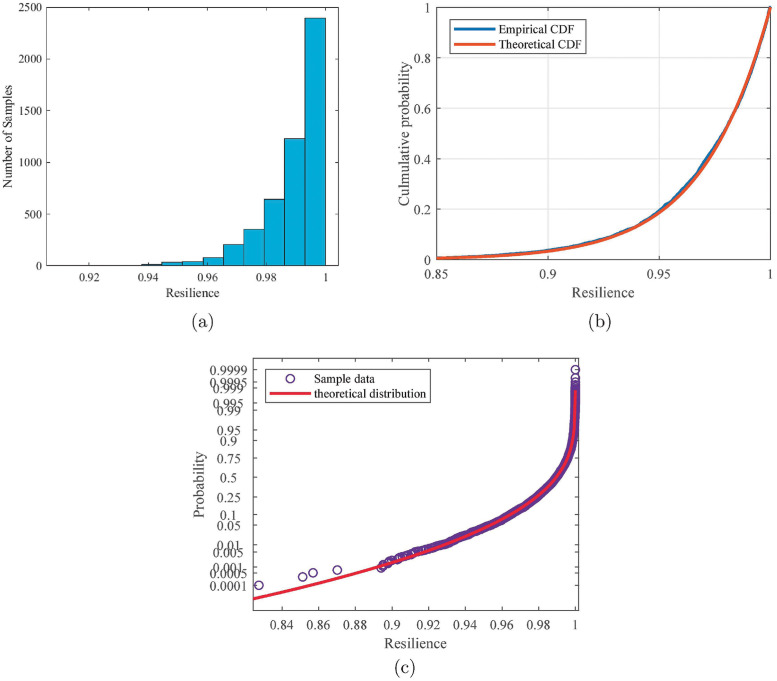
Statistics results for Experiments 1 in Table 2. a) histogram; b) CDF; c) probability plot (the fitted Weibull distribution is used as the theoretical function).

Experiment 1 in Table 2 is taken as an example to illustrate the RDIA process and results. Letting the number of simulations *n* be 5000, according to Eq 3, we can compute the simulation error as *ε* ≤ 0.08% with a confidence level of 1 − *α* = 95%. The histogram of the resilience samples obtained from the simulation is shown in Fig 4(a). Using the distribution fitting method, one can find that the system resilience obeys a Weibull distribution with the shape parameter *ξ* = −0.99988, scale parameter *σ* = 0.004564, and position parameter *μ* = 0.995432. From the empirical and theoretical CDF plots in Fig 4(b) and the probability plot in Fig 4(c), one can see that the empirical distribution curve of the resilience samples obtained in Experiment 1 is highly consistent with the fitting distribution curve, and only a slight deviation exists in some samples with low system resilience. Using a chi-squared test, the chi-squared statistic is computed to be *χ*^2^ = 9.3021. It is known that the critical value $\chi_{0.05}^{2}\left(6\right)=12.6$. Since $\chi^{2}\leq \chi_{0.05}^{2}$, we can conclude that the system resilience with the process functions and parameters in Experiment 1 obeys the Weibull distribution at a significance level of 5%.

We also find that all 18 experiments in Table 2 follow the Weibull distribution. The parameter estimation and the chi-squared test results of these 18 experiments are shown in Table 3. One can see that 13 chi-squared statistics in Table 3 satisfy a significance level *α* = 5%, 4 statistics satisfy *α* = 1%, and 1 statistic satisfies *α* = 0.5%.

**Table 3:** Distribution fitting results for experiments in Table 2.

No.	Shape parameter *ξ*	Scale parameter *σ*	Position parameter *μ*	Chi-squared statistic *χ*^2^
1	-0.99988	0.004564	0.995432	9.3021
2	-0.99815	0.007244	0.992736	9.1708
3	-1.01166	0.006619	0.993440	6.3915
4	-1.00473	0.013303	0.986747	11.3008
5	-1.00624	0.012333	0.987721	15.8348
6	-0.98600	0.015129	0.984641	6.5990
7	-0.97489	0.010014	0.989709	10.3277
8	-1.03237	0.017843	0.982695	5.6041
9	-0.99553	0.022594	0.977272	12.9884
10	-0.98317	0.022496	0.977099	14.7408
11	-1.07359	0.022177	0.979315	10.8050
12	-1.00270	0.02313	0.976913	7.7037
13	-1.02137	0.020399	0.980017	7.9690
14	-1.03697	0.03476	0.966436	8.4229
15	-1.02432	0.024886	0.975686	10.1542
16	-0.98392	0.026799	0.972732	8.9706
17	-1.02449	0.029377	0.97131	14.3174
18	-0.96725	0.121987	0.873491	17.1497

### Effects of performance process functions and parameters

#### Experiment design

Using the control variable method, we design 13 types of experiments to investigate the influence of the system performance process parameters on the resilience distribution. The specific parameters are shown in Table 4. Experiment Type 1 in Table 4 is the control group, and the other experiment types are experimental groups. Comparing the experimental results for Experiment Types 2–5, Experiment Types 6–9, and Experiment Types 10–13 with Experiment Type 1, we can investigate how the random variable *T*_*r*_, *T*_*d*_, and *Q*_*L*_ affect the resilience distribution, respectively.

**Table 4:** Experiments to find how the performance process parameters affect the resilience distribution.

Type	*Q*_*L*_	*T*_*d*_	*T*_*r*_
1	*P*(*Q*_*L*_ = 0.01*i*) = 0.01, *i* = 1, 2, …, 100	1/λ_*d*_ = 60 s	1/λ_*r*_ = 60 s
2			1/λ_*r*_ = 20 s
3			1/λ_*r*_ = 40 s
4			1/λ_*r*_ = 80 s
5			1/λ_*r*_ = 100 s
6	*P*(*Q*_1_ = 0.01*i*) = 0.01, *i* = 1, 2, …, 100	1/λ_*d*_ = 20 s	1/λ_*r*_ = 60 s
7		1/λ_*d*_ = 40 s	
8		1/λ_*d*_ = 80 s	
9		1/λ_*d*_ = 100 s	
10	*P*(*Q*_*L*_ = 0.01*i*) = 0.0125, *i* = 1, 2, …, 80	1/λ_*d*_ = 60 s	1/λ_*r*_ = 60 s
11	*P*(*Q*_*L*_ = 0.01*i*) = 0.016, *i* = 1, 2, …, 60		
12	*P*(*Q*_*L*_ = 0.01*i*) = 0.025, *i* = 1, 2, …, 40		
13	*P*(*Q*_*L*_ = 0.01*i*) = 0.05, *i* = 1, 2, …, 20		

Combining the 13 types of performance process parameters in Table 4 with the 3 system performance process functions in Eqs 1 and 2, we have 3 × 13 experiments. We use L/E/T to represent the performance processes that follow the linear/exponential/trigono -metric functions, respectively. We use a letter and a digit together to represent the experiment code, e.g., Experiment L1 is a performance process with a linear function and Type 1 parameters in Table 4. For the experiments with exponential functions in Table 4, letting *b* be *ln*(200), the experiments in Table 5 are added to discuss how the parameter *b* of the exponential function affects the system resilience distribution. Experiment E1 is used as the control group, and the other four experiments are experimental groups.

**Table 5:** Experiments to find the effect of *b* on the resilience distribution.

No.	*b*	*Q*_*L*_	*T*_*d*_	*T*_*r*_
E1(1)	*ln*(20000)	*P*(*Q*_*L*_ = 0.01*i*) = 0.01, *i* = 1, 2, …, 100	1/λ_*d*_ = 60 s	1/λ_*d*_ = 60 s
E1(2)	*ln*(2000)			
E1	*ln*(200)			
E1(3)	*ln*(20)			
E1(4)	*ln*(1.1)			

#### Discussion

**Fig 5:**
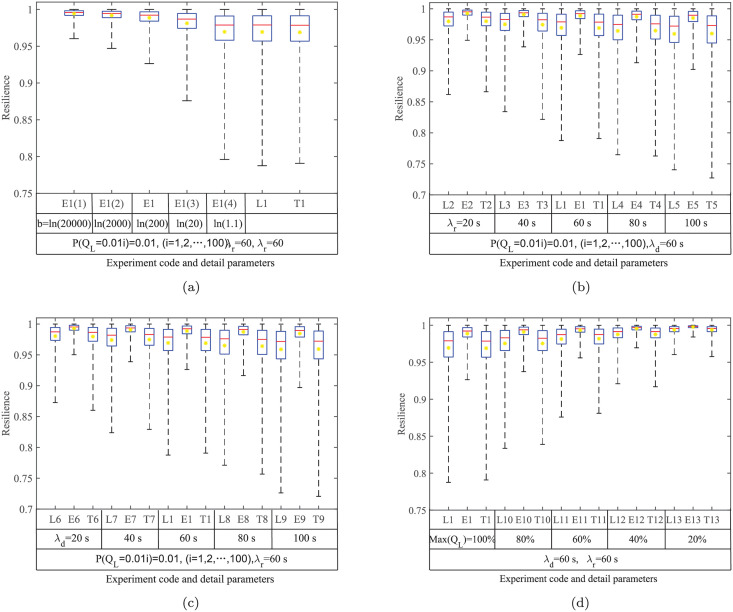
Sample box plots of experiments in Tables 4 and 5. a) effect of performance process functions; b) effect of *T*_*d*_; c) effect of *T*_*r*_; d) effect of *Q*_*L*_. The yellow dots represent the mean resilience $\bar{{\mathbb{R}}_{D}}$, the red lines represent the median resilience ${\mathbb{R}}_{D,0.5}$, the tops and the bottoms of the boxes represent the upper 75% percentiles ${\mathbb{R}}_{D,0.75}$ and lower 25% percentiles ${\mathbb{R}}_{D,0.25}$, respectively, and the top and bottom lines represent the maximum resilience ${\mathbb{R}}_{D,\text{max}}$ and minimum resilience ${\mathbb{R}}_{D,\text{min}}$, respectively.

The procedures for the experiments in Tables 4 and 5 are similar to those in Table 2, so we only discuss the experimental results here. Fig 5 provides the box plots of the system resilience with a parameter change.

**Fig 6:**
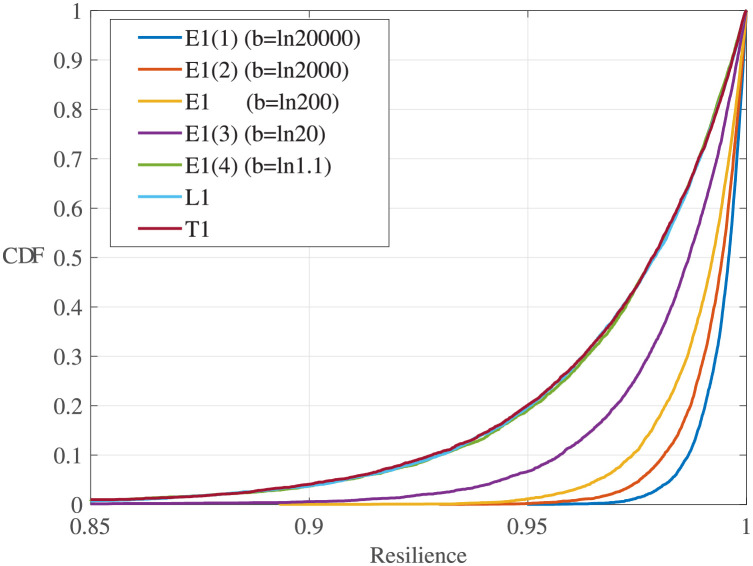
Sample CDFs of experiments in Tables 4 and 5.

**Fig 7:**
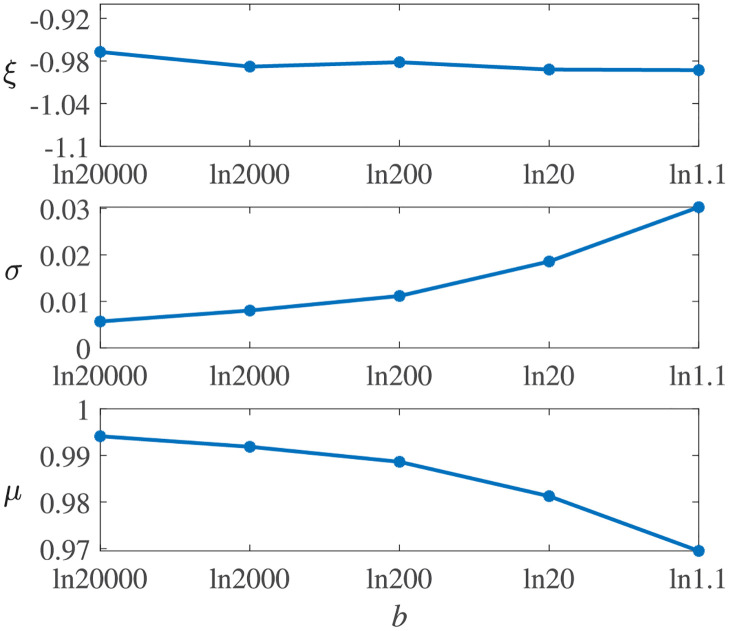
Parameters estimated of experiments in Table 5.

**Fig 8:**
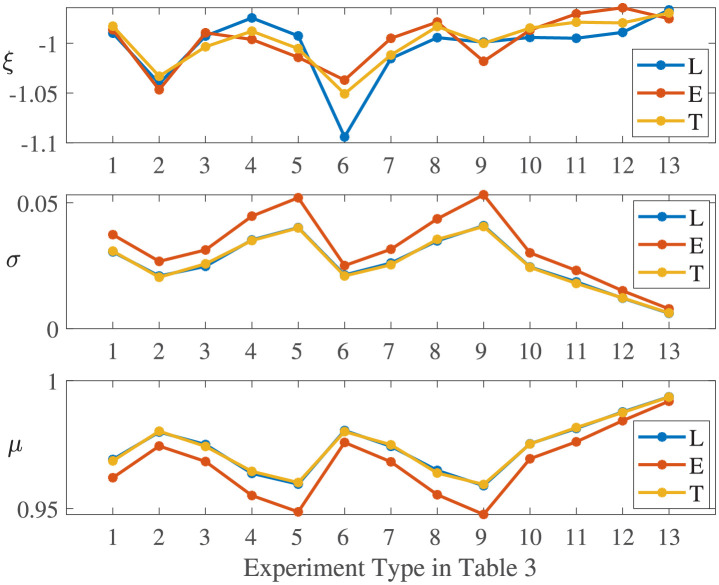
Parameters estimated of experiments in Table 4.

**Fig 9:**
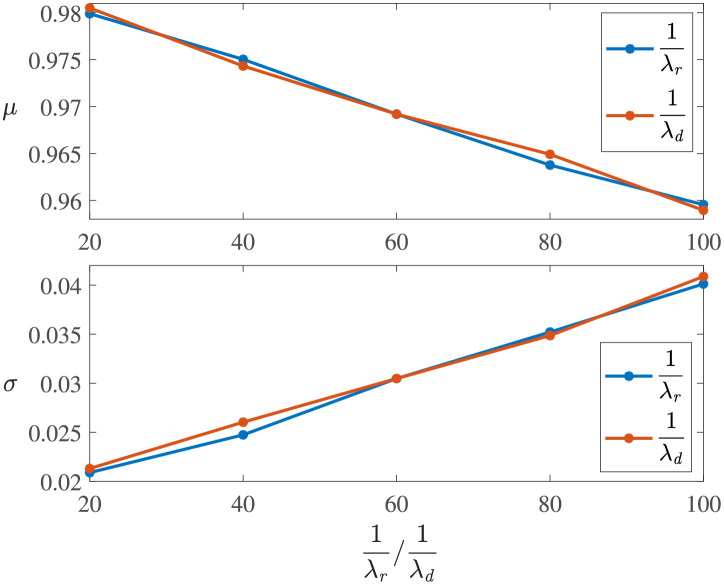
Estimated parameters of Experiments L1-L9.

**Fig 10:**
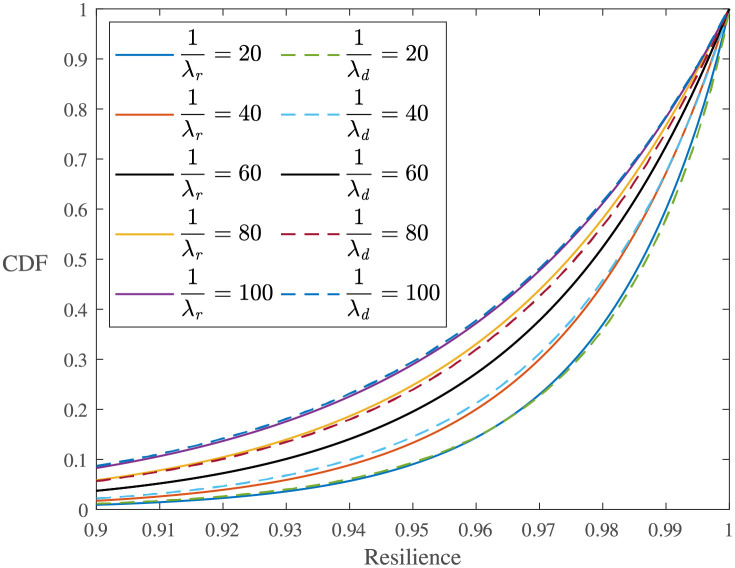
Sample CDFs of Experiments L1-L9.

**Fig 11:**
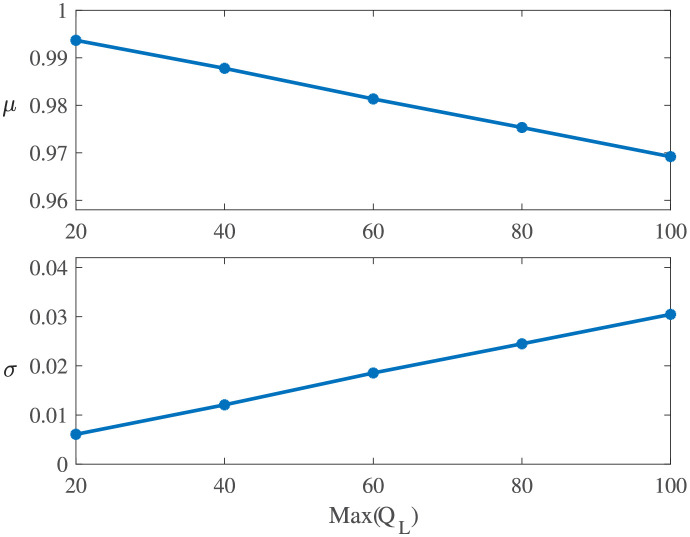
Estimated parameters for Experiments L1 and L10-L13.

From Fig 5, one can see that the medians of all the boxes are generally greater than the mean, and this indicates that the resilience distributions are right-skewed. This is because the system can return to the normal state within the maximum allowable recovery time *T*_*a*_ in most situations. It can also be found that the maximum resilience of all experiments is very close to 1. This is because the number of Monte Carlo iterations is large enough in each experiment, and there are always some processes with small performance degradations and short degradation/recovery durations. The results show that all the samples of the experiments in Tables 4 and 5 obey the Weibull distribution. The impacts of the performance process functions and parameters on the system resilience distribution are analyzed below:

1. **Effect of performance process functions**. According to Fig 5(a), the process functions affect the system resilience distributions. Fig 6 shows the sample resilience CDFs for all three types of resilience functions. Comparing the exponential resilience functions with different *b*, one can see that the CDF curve becomes steeper and most of the resilience samples are higher with a larger *b*. When *b* becomes closer to 0, the sample distribution becomes closer to those with linear and trigonometric resilience functions. This is because a small *b* indicates a small change rate of the exponential performance process, and in this case, the exponential function is close to the linear function.
  The parameters of the Weibull distributions obtained through distribution fitting for the experiments in Table 5 are shown in Fig 7. One can see that the shape parameter *ξ* does not change obviously with *b*, but a smaller *b* results in a smaller scale parameter *σ*, a larger position parameter *μ*, and a less resilient system.
Fig 8 compares the parameters estimated by fitting the Weibull distribution for the experiments in Table 4. One can see that the scale parameter *σ* and the position parameter *μ* are almost the same for experiments with linear and trigonometric performance process functions, and their shape parameters *ξ* are all near -1. Although the linear and trigonometric functions are different in curve shapes, their integrations within the same time period are similar, as shown in Fig 2. Moreover, the scale parameter *σ* of the experiments with exponential performance process functions is smaller than those for the other two types of functions, and the position parameter *μ* is larger than those for the other two types.
2. **Effect of *T*_*d*_ and *T*_*r*_**. Fig 5(b) and 5(c) shows the effect of both the degradation duration and the recovery duration on the system resilience distribution. One can see that the samples are more dispersed for the exponentially distributed *T*_*r*_ (or *T*_*d*_) with larger 1/λ. The system resilience decreases with the increase in the *T*_*r*_ (or *T*_*d*_) mean. This is because the longer the *T*_*r*_ (or *T*_*d*_) is, the longer the system operates at a low-performance level, resulting in lower resilience. The effects of *T*_*r*_ and *T*_*d*_ are almost the same since they both determine the duration of the system operating with degraded performance.
  The experiments with linear resilience functions are taken as an example. Their shape parameters *ξ* do not have significant laws, and their scale parameters *σ* and position parameters *μ* are shown in Fig 9. One can see that a larger *T*_*r*_ (or *T*_*d*_), a larger *σ* and a smaller *μ* results in a resilience distribution with a wider range.
Fig 10 shows the sample CDF of Experiments L1-L9. It can be seen that the effects of *T*_*d*_ and *T*_*r*_ on the resilience distributions are basically the same. This is because both *T*_*d*_ and *T*_*r*_ follow exponential distributions with the same parameter, and they jointly determine the time when the system operates at a degraded performance.
3. **Effect of *Q*_*L*_**. Fig 5(d) explores the effect of the maximum degradation performance *Q*_*L*_ on the system resilience distribution. One can see that the mean resilience increases with the decrease in the max(*Q*_*L*_), and the sample resilience is more concentrated with a smaller max(*Q*_*L*_). This phenomenon is inevitable because the maximum performance degradation *Q*_*L*_ directly determines the maximum performance that the system loses. Therefore, a smaller max(*Q*_*L*_) implies a larger system resilience. Taking experiments with linear resilience functions as an example, their estimated parameters are shown in Fig 11. One can see that a larger max(*Q*_*L*_) has a larger scale parameter *σ* and a smaller position parameter *μ*, indicating a lower system resilience.

## Case study

Here, a wireless end-to-end communication system under random electromagnetic interference is used as an example to explain our RDIA method with simulation or test data in practice. In this case, the bit error rate (BER) is chosen as the key performance index, and the maximum allowable recovery time is 12 minutes.

**Fig 12:**
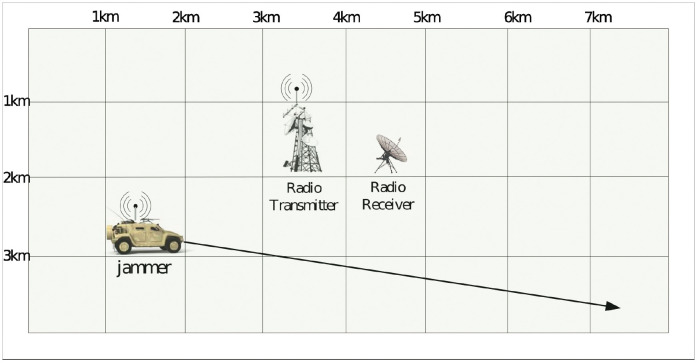
The end-to-end communication system under random electromagnetic interference.

The system consists of two fixed nodes, a transmitting one and a receiving one, and the distance between them is 1km. The transmitting node generates 1024-bit packets at a rate of 1.0 packets/second, and transmits them at 1024 bits/second over the channel. A jammer is applied to simulate the electromagnetic interference. It moves in a straight line at a constant speed within a 4*km* × 8*km* area as Fig 12 shows. Its speed follows a normal distribution, and its start and end coordinates follow uniform distributions. The parameters are shown in Table 6.

**Table 6:** Parameters of the jammer movement.

Parameters	Distribution
Speed (m/s)	*N*(10, 2^2^)
Start point *X*_*s*_ (km)	(*U*(0, 8), *U*(0, 8)
End point *X*_*e*_ (km)	(*U*(0, 4), *U*(0, 4))

**Fig 13:**
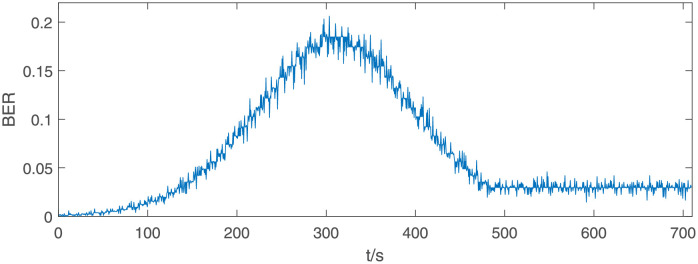
BER under the electromagnetic interference shown in Fig 12.

Let the simulation error be *ε* ≤ 0.08 with a confidence level of 1 − *α* = 95%, and the number of simulation iterations *n* is determined as 50 according to Eq 3. As it is not a numerical study, so we relax the requirements of simulation error. In each simulation run, we randomly generate the jammer trajectory according to the parameters shown in Table 6. The system BER is collected every 0.5 s during the simulation, and the system resilience of these samples are calculated using Eq 4. Fig 13 shows the BER under the electromagnetic interference shown in Fig 12. As the distance between the jammer and the receiving node is first far, then near, and later far, the system BER declines first, then rises, and ends up at a degraded level.

**Fig 14:**
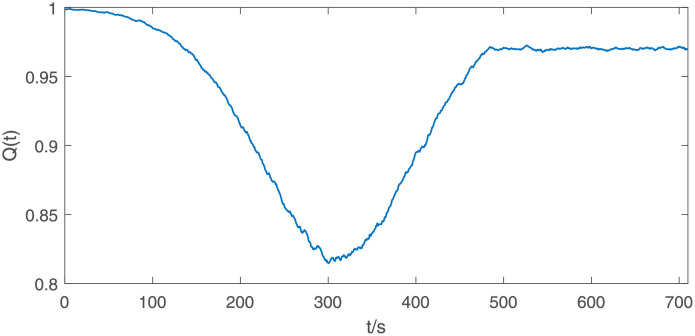
Normalized and smoothed performance under the electromagnetic interference shown in Fig 12.

**Fig 15:**
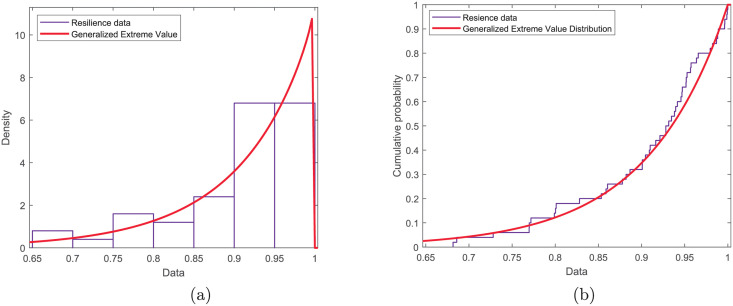
PDF and CDF of system resilience. a) PDF; b) CDF.

Since the BER is a smaller-the-better type parameter, we use the min-max normalization method to obtain the normalized performance data, and then smoothed it. Fig 14 shows the results obtained after the BER data in Fig 13 be normalized and smoothed. After calculating the system resilience under each electromagnetic interference, we fit the data using a generalized extreme value distribution with the shape parameter *ξ* = −1.012, scale parameter *σ* = 0.09541 and location parameter *μ* = 0.9056. The PDF and CDF of both the sample data and the fitting results can be seen in Fig 15. The chi-squared statistic is computed to be *χ*^2^ = 4.595. Comparing with the critical value $\chi_{0.05}^{2}\left(3\right)=7.815$ at 95% confidence level, we can draw the conclusion that the system resilience can be considered to obey the generalized extreme value distribution.

## Conclusion

Resilience reflects the ability of a system to withstand disruptions and quickly recover from them. It is an internal attribute of a system, as well as a random variable due to the randomness of both the disruption and the system’s response to it. To assist with system resilience design and analysis, this paper proposes a resilience distribution identification and analysis (RDIA) method to find the resilience distribution from a statistical perspective. Based on the functions and key parameters of the system’s performance processes after disruptions, this method uses the Monte Carlo method to obtain the resilience samples. Then the system resilience distribution can be determined using distribution identification, parameter estimation, and a goodness-of-fit test. Finally, the resilience distributions are analyzed for system resilience with typical performance processes. The results show that the resilience obeys the Weibull distribution. Our method needs system’s performance degradation/recovery function and related parameter distributions. In practice, it is not always easy to obtain such data. In this situation, we can try to collect several sets of system’s performance data that varies with time after disruptions. The case study shows how to inject disruptions and collect performance response data for system resilience distribution identification. Further, if no data can be used, classical resilience distribution types for typical performance processes can be used, and then the distribution type can be updated with data collection.

The contributions of our paper include the following:

1. A systematic method is proposed to identify the system resilience distribution based on the system performance processes after disruptions.
2. The resilience distributions are analyzed for typical performance degradation/recovery processes with linear, exponential, and trigonometric functions, as well as exponential distributed durations and discrete uniform distributed maximum performance degradation. The results show that the resilience obeys the Weibull distribution.
3. Our method aids understanding of system resilience from a statistical perspective, and the resilience distribution obtained can be further used for system resilience design and analysis.

Using our RDIA method, system resilience distribution can be obtained, and future studies will focus on more possible performance process types.

## Supporting information

S1 Data Experiments data files. (ZIP)
